# Transcriptomic signature of the follicular somatic compartment surrounding an oocyte with high developmental competence

**DOI:** 10.1038/s41598-017-07039-5

**Published:** 2017-07-28

**Authors:** Satoshi Sugimura, Norio Kobayashi, Hiroaki Okae, Tadayuki Yamanouchi, Hideo Matsuda, Takumi Kojima, Akira Yajima, Yutaka Hashiyada, Masahiro Kaneda, Kan Sato, Kei Imai, Kentaro Tanemura, Takahiro Arima, Robert B. Gilchrist

**Affiliations:** 1 Department of Biological Production, Tokyo University of Agriculture and Technology, Tokyo, 183-8509 Japan; 2 Laboratory of Animal Reproduction and Development, Graduate School of Agricultural Science, Tohoku University, Miyagi, 981-8555 Japan; 3 Department of Informative Genetics, Environment and Genome Research Center, Graduate School of Medicine, Tohoku University, Miyagi, 980-8575 Japan; 4 National Livestock Breeding Center, Fukushima, 961-8511 Japan; 5 Divition of Animal Life Science, Tokyo University of Agriculture and Technology, Tokyo, 183-8509 Japan; 6 Department of Sustainable Agriculture, Rakuno Gakuen University, Hokkaido, 069-8501 Japan; 7 Discipline of Obstetrics & Gynaecology, School of Women’s & Children’s Health, University of New South Wales, Sydney, 2052 Australia

## Abstract

During antral folliculogenesis, developmental competence of prospective oocytes is regulated in large part by the follicular somatic component to prepare the oocyte for the final stage of maturation and subsequent embryo development. The underlying molecular mechanisms are poorly understood. Oocytes reaching the advanced stage of follicular growth by administration of exogenous follicle-stimulating hormone (FSH) possess higher developmental competence than oocytes in FSH-untreated smaller follicles. In this study, the transcriptomic profile of the cumulus cells from cows receiving FSH administration (FSH-priming) was compared, as a model of high oocyte competence, with that from untreated donor cows (control). Ingenuity Pathway Analysis showed that cumulus cells receiving FSH-priming were rich in down-regulated transcripts associated with cell movement and migration, including the extracellular matrix-related transcripts, probably preventing the disruption of cell-to-cell contacts. Interestingly, the transcriptomic profile of up-regulated genes in the control group was similar to that of granulosa cells from atretic follicles. Interferon regulatory factor 7 was activated as the key upstream regulator of FSH-priming. Thus, acquisition of developmental competence by oocytes can be ensured by the integrity of cumulus cells involved in cell-to-cell communication and cell survival, which may help achieve enhanced oocyte-somatic cell coupling.

## Introduction

Signals from the somatic cell compartment of ovarian follicles, such as from granulosa and cumulus cells, regulate oocyte competence; defined as the capacity to support fertilization, pre-implantation phases of embryo development, and full-term development^1^. The somatic cell compartment is under the control of gonadotropins (follicle-stimulating hormone [FSH] and luteinizing hormone [LH]) during folliculogenesis, which interact with local growth factors and steroids^1^. The success of modern day *in vitro* fertilisation (IVF) is highly dependent on the administration of FSH to women. This leads to the development of multiple follicles allowing the retrieval of oocytes that would otherwise not be developmentally viable. Likewise, women undergoing oocyte *in vitro* maturation (IVM) usually receive FSH injections prior to oocyte retrieval^2^. Hence, it is important to understand the impact of exogenous FSH of the molecular functioning of the follicle and how this regulates oocyte developmental competence.

During antral folliculogenesis, prior to the surge in gonadotrophin levels, FSH binds to the FSH receptor and modifies follicular somatic cells, which participate in acquisition of oocyte competence, meiotic maturation and ovulation. The expression of LH and epidermal growth factor (EGF) receptors on follicular somatic cells are well-characterized actions exerted by FSH^3, 4^. Furthermore, FSH increases gap junctional communication (GJC) between follicular somatic cells and between the oocyte and somatic cells^5^, probably via cyclic adenosine monophosphate (cAMP)-phosphate kinase A (PKA) signalling^6^. GJC enables the passage of cAMP, cyclic guanosine monophosphate (cGMP), metabolites, exosomes, and potentially RNA into the oocytes from follicular somatic cells and between somatic cells, which play a crucial role in the regulation of meiosis and oocyte competence^7, 8^. During the ovulatory cascade, expression of EGF-like peptides such as amphiregulin (AREG), epiregulin, and betacellulin on mural granulosa cells is induced in rapid response to the FSH and LH surges, and then the EGF-like peptides activate the EGFR on cumulus cells^9–11^. EGFR signalling stimulates gene expression that enables cumulus expansion, in cooperation with the potent oocyte-secreted factors (OSFs), in particular, bone morphogenetic protein 15 (BMP15), growth differentiation factor 9 (GDF9), and the BMP15/GDF9 heterodimer cumulin^12–14^, which is essential for ovulation and oocyte capture by the infundibulum.

Oocytes gradually acquire developmental competence during folliculogenesis^15^. Hence, oocytes from small antral follicles have low competence to reach the blastocyst stage compared with oocytes from large follicles^16–19^. In a pig experimental model, the COCs derived from small antral follicles possess less competence for cumulus expansion in response to EGF or EGF-like peptides because of immature EGFR signalling in cumulus cells^20–22^. This is a reason why the oocytes from small antral follicles have low developmental competence. Hence, promotion of EGFR signalling in cumulus cells might be a key component in the acquisition of oocyte developmental competence^20^. On the other hand, in mouse model, the cumulus cells derived from small follicles (non-gonadotropin primed) are competent to undergo expansion *in vitro*, in a manner similar to that of cumulus cells derived from large antral follicles following gonadotropin priming^23, 24^. Despite this morphological similarity, there was a transcriptomic difference in the cumulus cells from small and large follicles. In particular, the cumulus cells from large follicles were enriched in transcripts that regulate metabolism, cell differentiation, and adhesion^23^. This result suggests that not only EGFR signalling but also other network signalling in cumulus cells participate in the development of oocyte competence.

The proportion of bovine oocytes exhibiting developmental competence greatly increases in follicles >8 mm (large antral). Some oocytes in 3 mm follicles (small antral) have acquired a degree of developmental competence, but require additional “pre-maturation” *in vivo* prior to final maturation by the surge in gonadotrophin levels to induce competence^25^. Pre-maturation *in vivo* can be driven by advancing follicular growth with FSH administration; the oocytes derived from cows subjected to FSH treatment prior to ovum pick up (OPU) have higher developmental competence than those derived from untreated cows^26, 27^. Thus, various signalling pathways may be changed by advanced follicular growth implicated in subsequent oocyte development. Our hypothesis is that the transcriptomic landscape in cumulus cells is changed during follicular growth induced by FSH administration. The transcriptomic landscape offers the molecular and functional features of the somatic cell components surrounding oocytes with high developmental competence, which will contribute to the regulation of “oocyte capacitation”.

In the present study, our aim was to analyse the transcriptomic profile of cumulus cells surrounding highly competent oocytes. First, we examined the effect of FSH-priming consisting of FSH administration in the absence of a dominant follicle, on *in vitro* blastocyst formation of OPU-derived oocytes. Our results indicate that the oocytes derived from cows subjected to FSH-priming possess higher oocyte developmental competence compared to those from untreated cows (control). Hence, we used the COCs derived from cows subjected to FSH-priming as a high competence model. We then analysed the transcriptome of cumulus cells from the control and FSH-priming groups by RNA sequencing (RNA-seq) and real-time RT-PCR.

## Results

### FSH-priming prior to oocyte retrieval enhances developmental competence of oocytes matured with gonadotrophin

**Figure 1:**
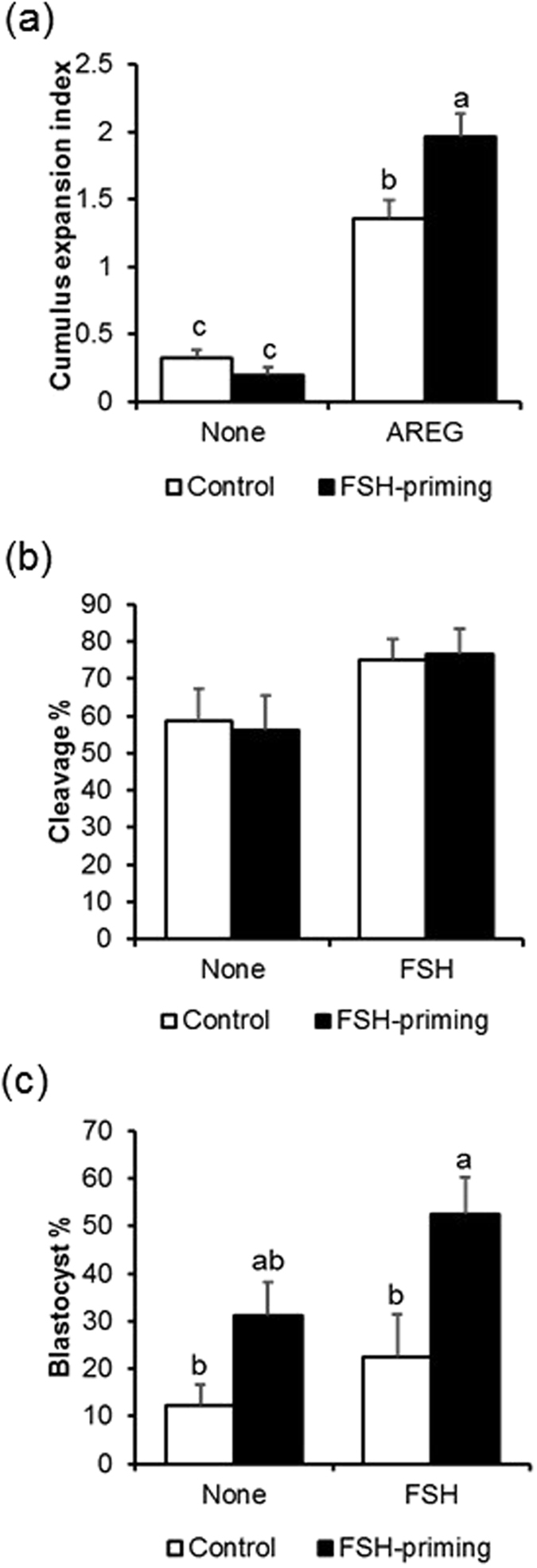
Extracellular expansion of the cumulus cells and developmental competence of oocytes from both unpriming and FSH priming cows. The cumulus expansion index of the COCs derived from donor cows without (control) or with FSH-priming, which were cultured without the ligand (none) or with amphiregulin (AREG), was examined at 22 h of oocyte maturation (**a**). Effect of FSH-priming prior to OPU and supplementation of FSH during *in vitro* maturation on oocyte developmental competence were examined by measuring the following on-time embryo development milestones; cleaved embryos at day 2 (**b**) and blastocyst development on day 7 (**c**).

Although there were no changes in the total number of follicles, a significantly higher number of large antral follicles (>8 mm) and lower number of small antral follicles (2–4 mm) were observed in cows following FSH-priming (P < 0.05) (Table 1). The COCs were morphologically similar between the control and FSH-priming groups (Table 2). The acquisition of EGF responsiveness by cumulus cells is a milestone of oocyte developmental competence^20^. To confirm EGF-like peptide responsiveness, we measured the cumulus expansion of COCs cultured with AREG between the control and FSH-priming groups. In both groups, cumulus expansion was induced by AREG; however, the level was significantly higher in the FSH-priming group (P < 0.05) (Fig. 1a). This result indicates that COCs from cows subjected to FSH-priming are more responsive to EGF ligands, than COCs from control cows. Post maturation and fertilization, there was no significant difference in embryo cleavage across all groups (Fig. 1b), suggesting oocytes derived from control and FSH-priming groups have similar competences on oocyte maturation and fertilization. In the presence of FSH in oocyte maturation medium, FSH-priming group increased blastocyst development by 2.3-fold, compared to the control group (control: 22.5%, FSH-priming: 52.5%; P < 0.05). However, in the absence of FSH in oocyte maturation medium, there was no significant difference between FSH-priming and control groups on blastocyst development (control: 12.2%, FSH-priming: 31.2%; Fig. 1c). Furthermore, blastocyst development was observed in all 12 cows subjected to FSH-priming when the COCs were matured with FSH, suggesting that the combined use of FSH-priming and FSH during the oocyte maturation stage yields maximum oocyte competence. Based on the result, COCs exposed to FSH-priming are defined as a high oocyte developmental competence model.

**Table 1:** Effect of FSH-priming on follicle size.

Treatment	No. of follicles (mean ± SD)			
	Total	2 to 4 mm	5 to 8 mm	>8 mm
Control	17.0 ± 6.5	14.3 ± 6.8^a^	0.8 ± 0.5	2.0 ± 0^b^
FSH-priming	20.8 ± 10.3	2.5 ± 2.5^b^	0.5 ± 1	17.8 ± 10.4^a^

^a,b^Different letters indicate significant difference at P < 0.05.

**Table 2:** Effect of FSH-priming on morphological quality of COCs.

Treatment	No. of COCs of different categories (mean ± SD of four replicates)				
	A	B	C	D	A + B
Control	5.7 ± 7.5	5.8 ± 3.6	2.3 ± 2.2	5.5 ± 4.2	11.5 ± 6.6
FSH-priming	8.5 ± 5.9	8.3 ± 3.3	0.3 ± 0.5	2.0 ± 3.4	16.8 ± 8.0

### Biofunctions in the cumulus cells from cows subjected to FSH-priming

**Figure 2:**
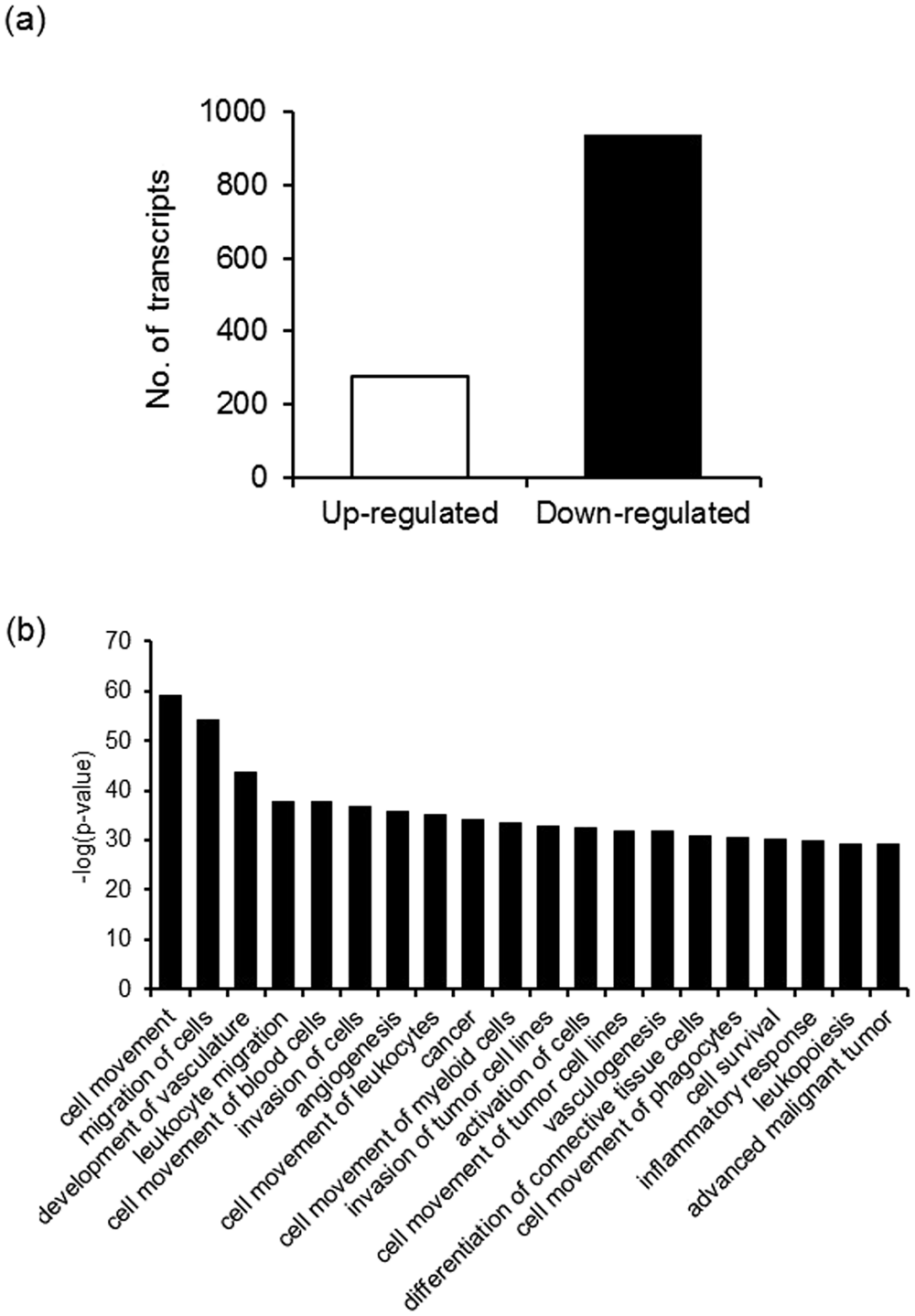
FSH-priming-sensitive transcripts and biological function in bovine cumulus cells. Number of FSH-priming-sensitive transcripts were defined as (FSH-priming_FRKM + 0.1)/(Control_FRKM + 0.1) ≥ 2 and FSH-priming_FRKM ≥ 1 (up-regulated) and (FSH-priming_FRKM + 0.1)/(Control_FRKM + 0.1) ≤ 0.5 and Control_FRKM ≥ 1 (down-regulated) (**a**). FSH-priming-sensitive biological functions were identified by Ingenuity Pathway Analysis (IPA). The top 20 functions for which an activation z score was ≤ −2.0 and −log(p-value) > 1 are shown as decreased biofunctions (**b**). The functions were identified and ranked by their −log(p-value). The significance of functions was defined as −log(p-value) > 1.

In this study, 1216 transcripts were identified as FSH-priming-sensitive genes. Compared to control, 937 transcripts were down-regulated and 279 were up-regulated in the FSH-priming group, relative to controls, suggesting a tendency towards global suppression of cumulus cell transcription in the FSH-priming group (Fig. 2a and Supplementary Table S3). Cell movement, migration of cells, and development of vasculature identified as the top three biofunctions in the FSH-priming group (Fig. 2b). Cell movement and migration involved 338 and 305 molecules, respectively, which included well known cumulus cell extracellular matrix-related genes such as pentraxin 3 (*PTX3*), tumour necrosis factor alpha-induced protein 6 (*TNFAIP6*), secreted phosphoprotein 1 (*SPP1*), and hyaluronan synthase 2 (*HAS2*) as the top molecules decreased in the FSH-priming (Fig. 2b and Supplementary Table S4). Morbidity mortality, organism death, and glucose metabolism disorder were identified as the top three biofunctions to undergo an increase (Supplementary Table S4). Morbidity or mortality biofunctions included interferon regulatory factor 7 (*IRF7*), interferon-stimulated gene 15 (*ISG15*), and signal transducers and activator of transcription 1 (*STAT1*) as increased target molecules in FSH-priming (Supplementary Table S4).

### Upstream regulators and canonical pathways of cumulus cells from cows subjected to FSH-priming

**Figure 3:**
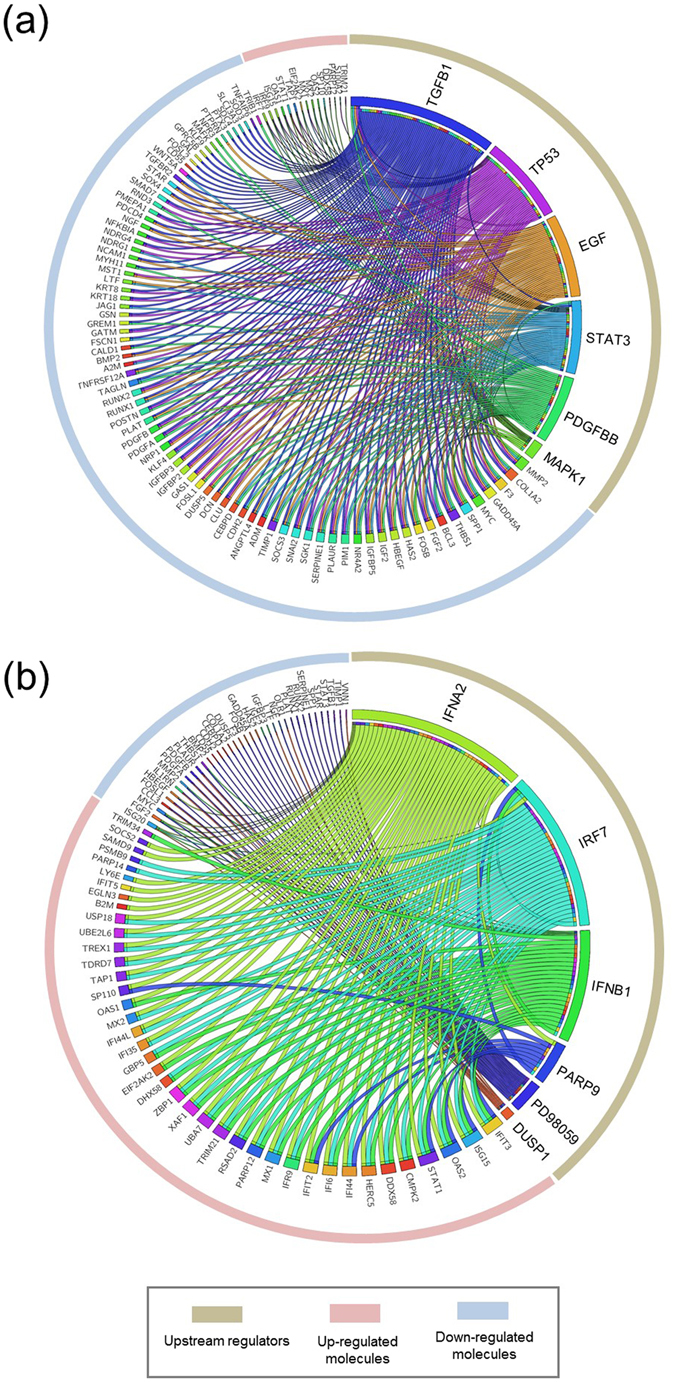
Upstream regulator analysis of FSH-priming-sensitive transcripts in cumulus cells. Ingenuity Pathway Analysis was used for the prediction of upstream molecules including cytokines, growth factors, transcription factors and chemicals. Selected upstream molecules, from the top 30, whose predicted activation status was “inhibited” (**a**) or “activated” (**b**) with corresponding molecules are shown. Inhibited and activated upstream molecules were defined as an activation z-score ≤ −1.5 and −log(P-value) > 1, and as an activation z-score ≥ 1.5 and −log(p-value) > 1, respectively. The upstream molecules were ranked by their −log(p-value). The significance was defined as −log(p-value) > 1. Brown, pink, and blue lines indicate upstream regulators, and up-regulated and down-regulated genes stimulated by each upstream molecule, respectively.

**Figure 4:**
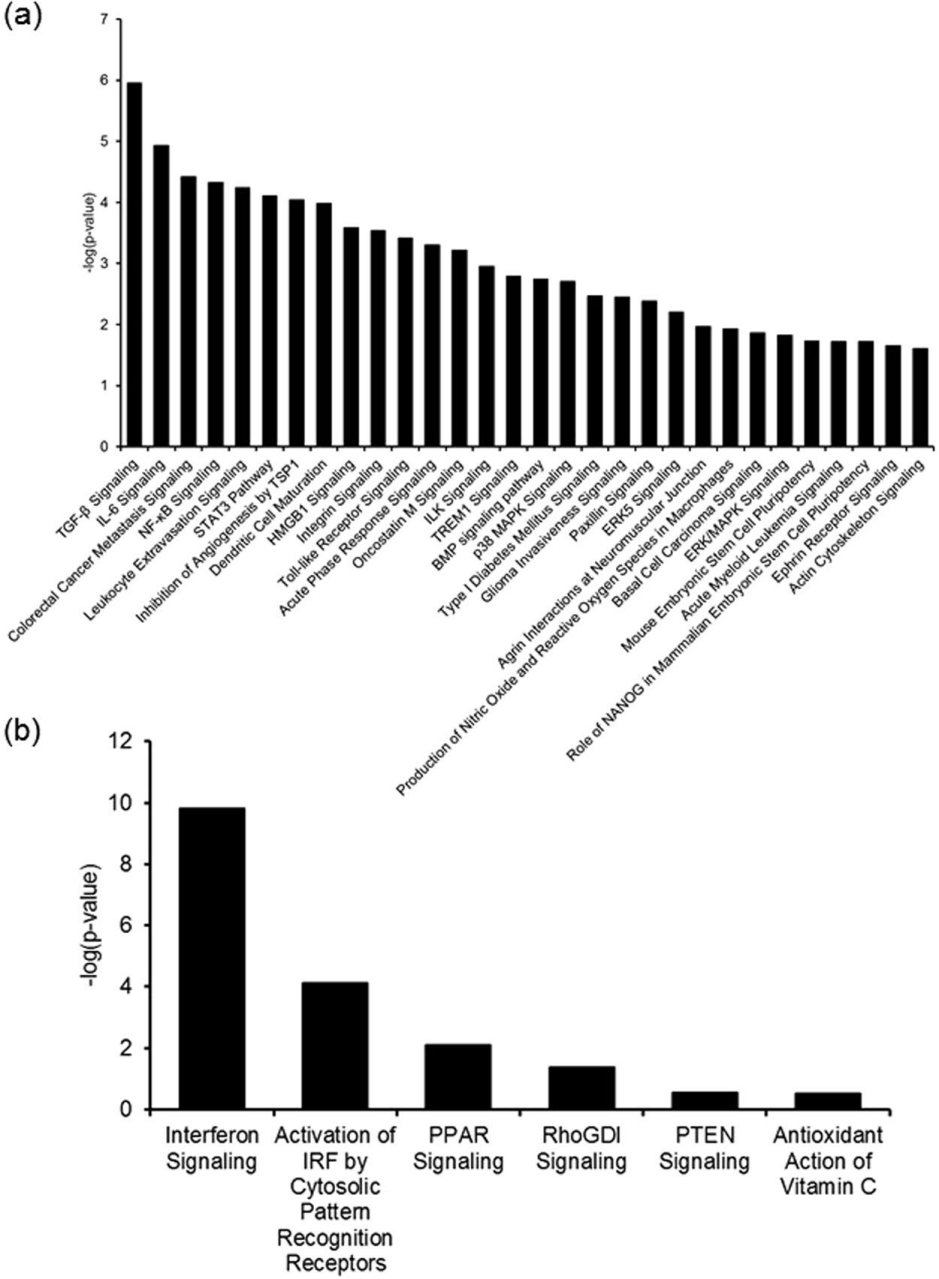
Canonical pathway analysis in bovine cumulus cells with FSH-priming. Ingenuity Pathway Analysis was used for predicting canonical pathways. The canonical pathways are identified and ranked by their z-score and -log(*P*-value). Down-regulated (**a**) and up-regulated canonical pathways (**b**) by FSH-priming were defined as a z-score ≤ −1.5 and −log(*P*-value) > 1, and as a z-score ≥ 1.5 and −log(*P*-value) > 1, respectively. The significance of canonical pathway was defined as −log(*P*-value) > 1.

Transforming growth factor beta 1 (TGFB1), STAT3, tumour protein p53 (TP53), platelet-derived growth factor BB (PDGFBB), mitogen-activated protein kinase 1 (MAPK1), and EGF, predicted as the top 30 upstream regulators undergoing inhibition, are shown in Fig. 3a and Supplementary Table S4. *PTX3*, *TNFAIP6*, *SPP1*, *HAS2*, matrix metalloproteinase 2 (*MMP2*), and thrombospondin 1 (*THBS1*) were included as the target molecules of TGFB1. Interleukin 6 (IL6) and nuclear factor kappa B (NF-_K_B) are also listed in the top 30 upstream regulators. Based on these results, TGFB signalling, STAT3 pathway, IL6 signalling, and NF-_K_B signalling were included as canonical pathways down-regulated by FSH-priming (Fig. 4a and Supplementary Table S6). In addition to these canonical pathways, dendritic cell maturation, inhibition of angiogenesis by thrombospondin 1 (TSP1), and p38 mitogen-activated protein kinases (p38 MAPK) were identified. On the other hand, interferon alpha 2 (IFNA2), IRF7, interferon beta 1 (IFNB1), poly [ADP-ribose] polymerase 9 (PARP9), and dual specificity protein phosphatase 1 (DUSP1) were predicted as activated upstream regulators in FSH-priming (Fig. 3b and Supplementary Table S5). *ISG15*, *IRF7*, 2′-5′-oligoadenylate synthetize 1 (*OAS1*), and the interferon-induced GTP-binding protein Mx1 (*MX1*), which are interferon-stimulated genes, were included as the up-regulated target molecules of IFNA2. Conversely, *HAS2*, *TGFB1*, *THBS1*, *HBEGF*, *PDGFB* are the down-regulated target molecules of PD98059. Interferon signalling was identified as top of up-regulated canonical pathways in the FSH-priming group (Fig. 4b and Supplementary Table S6). These results suggest that activation of interferon signalling and inhibition of MEK signalling in cumulus cells were induced by FSH-priming.

### Follicular atresia is suppressed by FSH-priming

The population of atretic follicles before the LH surge is 50–80% of all follicles on the basis of stereomicroscopic evaluation, apoptosis, and oestradiol- and insulin growth factor-binding proteins (IGFBPs), suggesting that a relatively large population of follicles before the LH surge could be unhealthy^28^. To evaluate the health status of cumulus cells derived from the control and FSH-priming groups at the transcriptomic level, RNA-seq data were compared with the transcriptomic profile of granulosa cells of bovine ovarian atretic follicles, as reported by Hatziodos *et al*.^29^ Table 3 presents a profile of up-regulated genes in the control group compared with the FSH-priming group which significantly overlapped with the genes from the granulosa cells of atretic follicles, including *THBS1* and *MMP2*. Furthermore, the profile of up-regulated genes in the FSH-priming group significantly overlapped with the genes from healthy granulosa cells, including the cytochrome p450 family 19 subfamily A (*CYP19A1*).

**Table 3:** Similarity of gene expression profile between granlulosa cells in atretic follicles and cumulus cells in unstimulated follicles.

Genes	Genes of present RNA-seq data	Overlapped genes to Hatzirodos *et al*. BMC Genomics (2014)							
		Fold change ≥ 3 & P < 0.05		Fold change > 4 & P < 0.05		Fold change ≥ 3 & P < 0.005		Fold change > 4 & P < 0.005	
		Healthy > Atresia	Hetalthy < Atresia	Healthy > Atresia	Hetalthy < Atresia	Healthy > Atresia	Hetalthy < Atresia	Healthy >Atresia	Hetalthy < Atresia
FSH-priming > Control^a^	279	20**	6	10**	4	19**	6	10**	4
FSH-priming < Control^b^	937	9	218**	4	160**	8	213**	4	158**
Total	26372	444	576	160	316	414	562	154	313

^a^(FSH-priming_FRKM + 0.1)/(Control_FRKM + 0.1) ≥ 2 and FSH-priming_FRKM ≥ 1. ^b^(FSH-priming_FRKM + 0.1)/(Control_FRKM + 0.1) ≤ 0.5 and Control_FRKM ≥ 1. **Present RNA-seq data significantly overlapped to the data of Hatzirodos *et al*. at *P* < 0.01.

### Validation of FSH-priming-sensitive transcripts

**Figure 5:**
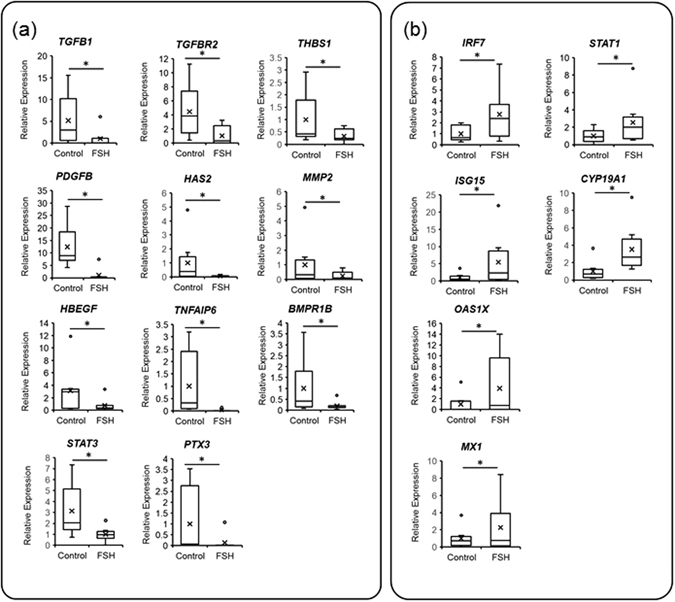
Validation of RNA-seq results by qPCR. Selected down-regulated (**a**) and up-regulated FSH-priming-sensitive genes (**b**) in bovine cumulus cells are shown. mRNA expression levels in the cumulus cells derived from eight donor cows without (control) or with FSH-priming (FSH) are represented as a box-and-whisker plots. Boxes reflect two quartiles, the 25th and 75th percentiles, and the interior horizontal line indicates the median. Whiskers indicate the maximum and minimum values within the acceptable range defined by the two quartiles. Open circles denote outliers. Crosses indicate mean value. Asterisks indicate significant difference at *P* < 0.05. *TGFB1*, transforming growth factor beta 1; *PDGFB*, platelet-derived growth factor subunit B; *HBEGF*, heparin-binding epidermal growth factor-like growth factor; *STAT3*, signal transducers and activator of transcription 3; *TGFBR2*, transforming growth factor beta receptor 2; *HAS2*, hyaluronan synthase 2; *TNFAIP6*, tumour necrosis factor, alpha-induced protein 6; *PTX3*, pentraxin 3; *THBS1*, thrombospondin 1; *MMP2*, matrix metalloproteinase 2; BMPR1B, bone morphogenetic protein receptor type 1B; *IRF7*, Interferon regulatory factor; ISG15, interferon-stimulated gene 15; *OAS1X*, 2′-5′-oligoadenylate synthetase 1, 40/46 kDa; *MX1*, interferon-induced GTP-binding protein; *STAT1*, signal transducers and activator of transcription 3; *CYP19A1*, cytochrome p450 family 19 subfamily a member 1.

To validate differentially expressed genes in the RNA-seq data, we analysed the mRNA level by real-time RT-PCR of 17 selected FSH-priming-sensitive genes (Fig. 5). Genes examined that were putatively down-regulated by FSH-priming included (Fig. 5a): the upstream regulators *TGFB1*, *PDGFB*, *HBEGF*, *and STAT3*; TGFB receptor 2 (*TGFBR2*) as the receptor of TGFB1; *HAS2*, *TNFAIP6* and *PTX3* as the extracellular matrix-related genes*;* and *THBS1*, *MMP2*, *and BMPR1B* as atretic markers^29^. Upregulated genes examined included (Fig. 5b): *IRF7* as an upstream regulator; *ISG15*, OAS1 40/46 kDa (*OAS1X*), *MX1*, *STAT1* as interferon signalling-related genes; and *CYP19A1* as a marker of healthy large follicles^30, 31^. The expression of all genes significantly differed between the control and FSH-priming groups (P < 0.05).

## Discussion

Oocyte competence to support embryo development is regulated by somatic cells surrounding the oocyte. These somatic cells are substantially modified by gonadotrophins during folliculogenesis. From a practical perspective, exogenous FSH treatment before OPU enhances bovine oocyte developmental competence^27, 32^. Furthermore, in mouse and human studies, equine chorionic gonadotropin/FSH-priming before oocyte retrieval enhance oocyte developmental competence during preimplantation embryo development and also to term^33, 34^. Global changes in follicle development and function are responsible for the improved subsequent oocyte quality and, due to their intimate association with the oocyte, the cumulus cells transmit and/or are responsible for these key changes in the oocyte during the final stages of oocyte development. As such developmental changes are critical to healthy early development, understanding the underlying molecular mechanisms in follicular somatic cells such as cumulus cells is important^23^. Here, we showed that FSH-priming drastically modulates gene expression in the cumulus cells, in particular in relation to increasing cell-to-cell communication and anti-inflammatory response, which may be implicated in the acquisition of oocyte developmental competence. This is the first report that describes the effect of FSH-priming on the RNA-seq profile of cumulus cells. This finding is likely to be important in efforts to enhance oocyte developmental competence not only *in vivo*, but also in *in vitro* maturation systems such as assisted reproduction technologies (ART).

The final stage of oocyte maturation and ovulation is mediated by EGF-like peptides stimulated by the surge in gonadotrophins. Functional EGFR signalling is developmentally regulated in the ovarian follicles, and the acquisition of somatic cell signalling capability is likely to be an important developmental milestone for oocytes^20^. In pigs, oocytes in growing small antral follicles with inherently low developmental competence are poor responders to EGF or EGF-like peptides^20–22^. In the current study, in a bovine model, although the COCs from small antral follicles (control group) were responsive to EGF-like peptides in terms of cumulus expansion, the extent of responsiveness was higher in the COCs from large antral follicles following FSH-priming, despite the fact that EGFR expression did not differ between the control and FSH-priming groups (Table S3). Promoting the responsiveness of cumulus cells to EGF-like peptides increases oocyte developmental competence^22^. Previously, we have suggested that improved oocyte competence may be aided by EGF-like peptide-based stimulation of cumulus cells by facilitating oocyte mitochondrial activity and energy production required for subsequent development^8, 35^. Furthermore, EGF-like peptides stimulate RNA translation in matured oocytes, which is critical for embryo development^36^. The developmental competence of oocytes matured *in vitro* with FSH was increased by FSH-priming. The pre-maturation *in vivo* of cumulus cells subjected to FSH-priming may allow efficient production of nutritional molecules, thanks to the surge in gonadotrophin levels via a functional EGFR signalling network.

**Figure 6:**
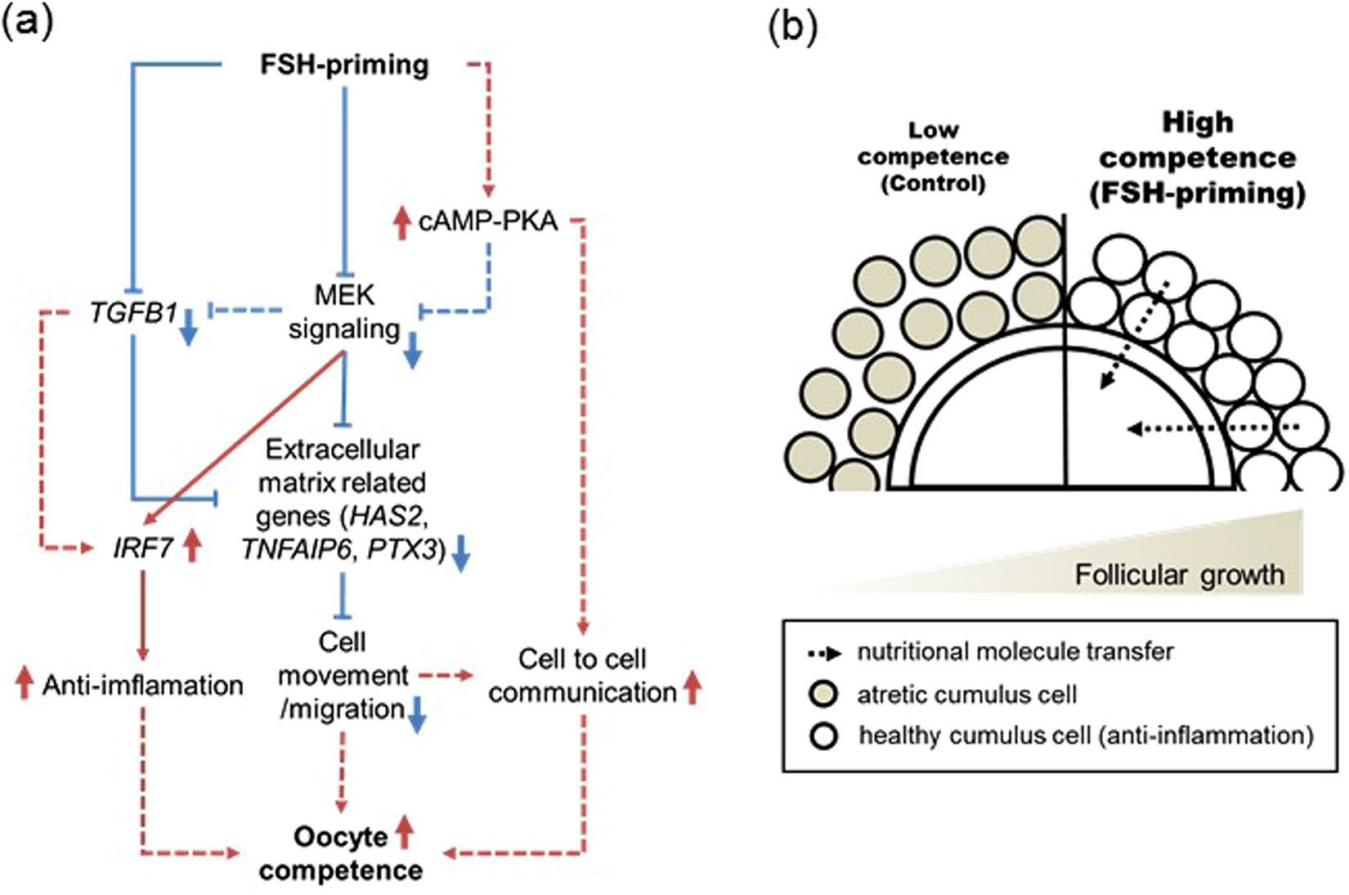
Hypothetical signalling network and status in bovine cumulus cells modified by FSH-priming based on RNA-seq expression signature. FSH-priming stimulates an anti-inflammatory cascade via the activation of IRF7 pathway following TGFB1 pathway inhibition. The inhibited TGFB1 pathway may result from the inhibition of MEK signalling. FSH-priming prevents cell movement/migration via the inhibition of genes involved in extracellular matrix expansion (HAS2, TNFAIP6, and PTX3) following MEK signalling inhibition, which may be related to enhancing cell-to-cell communication. Moreover, FSH PRIMING stimulates cAMP-PKA, which may participate in the inhibition of MEK signalling and activation of cell-to-cell communication such as gap-junctional communication (**a**). Cumulus cells in a low competence model without FSH-priming are in a state of poor cell-to-cell communication, progressing atresia, and driving a spontaneous ovulation-like cascade. Conversely, cumulus cells in a high competence model following FSH-priming are in a state of enhanced cell-to-cell communication and are maintained healthy by the activated anti-inflammatory cascade, which allow the transport of nutritional molecules from cumulus cells to oocytes at the time of final oocyte maturation (**b**). MEK, mitogen-activated protein kinase/extracellular signal-regulated kinase kinase; cAMP-PKA, cyclic adenosine monophosphate-protein kinase A signalling pathway.

Analysing the transcriptomic landscape in the cumulus cells from small (low oocyte competence) and large antral follicles (high oocyte competence) may provide more details about the underlying functional and molecular changes which participate in the development of competence in oocytes during antral folliculogenesis. RNA-seq analysis showed drastic transcriptomic changes (change in 1274 genes) in the cumulus cells subjected to FSH-priming; however, down-regulated genes constituted a major population of the altered transcriptomic profile, contrary to our expectation. These down-regulated genes were associated with cell movement and migration, and included extracellular matrix-related genes such as *HAS2*, *TNFIP6*, and *PTX3*. These genes are characteristically up-regulated in cumulus cells during oocyte maturation, and this is also associated with enhanced oocyte competence^37, 38^, however it should be noted that in the current study, cumulus cells were collected immediately prior to this stage (i.e. from immature unexpanded COCs). As a well-characterised action, expansion of the extracellular matrix during oocyte maturation, which involves the movement/migration of cumulus cells within the matrix, disrupts oocyte-cumulus and cumulus-cumulus GJC. Bidirectional communication between the oocyte and cumulus cells via GJC is essential for enabling the transfer of nutrients and other small molecules, along with ensuring that the oocyte acquires the molecular machinery required to support embryo development. Hence, an important feature of follicular growth treatment associated with improved oocyte quality may be to prevent the disruption of cell-to-cell communication, such as GJC, in the immature COC (Fig. 6a).

Activation of cAMP-PKA signalling is one of the reasons for the decrease in cell movement/migration within cumulus cells in cows subjected to FSH-priming. A recent study showed that cAMP, as the key secondary messenger of FSH, suppresses ERK1/2, which is part of the MAPK system^39^. Cyclic AMP stimulates a decrease in ERK1/2 phosphorylation via DUSP1 phosphatase, consequently causing the down-regulation of extracellular matrix-related genes such as *HAS2*. In the present data obtained by IPA, MAPK1, also known as ERK2, was predicted to be an inhibited upstream regulator. On the other hand, DPSP1 and the inhibitor of MEK1/2, which is the kinase of ERK1/2, were predicted as activated upstream regulators. Furthermore, *HAS2* was included as the target molecule of the inhibitor of MEK1/2. Thus, decreased cell movement and migration may be implicated in the inhibition of extracellular matrix expansion in cumulus cells, which could be induced by the negative regulation of MEK signalling, probably via cAMP-PKA signalling (Fig. 6a). FSH also stimulates enhancement in GJC, at least in part by a cAMP-PKA-based mechanism^5^. Using cAMP-elevating agents such as forskolin and IBMX for *in vitro* pre-maturation, GJC is enhanced and helps improve oocyte competence^40^. The enhanced GJC affects oocyte chromatin remodelling and transcription^41^, oocyte metabolism^8^, and accumulation of intra-oocyte GSH^42^, which are necessary for the acquisition of developmental competence. Thus, enhancement of GJC by elevating and/or sustaining cAMP concentration in cumulus cells subjected to FSH-priming may also be a reason for improved oocyte competence.

Cumulus cells subjected to FSH-priming were enriched in transcripts associated with interferon signalling and IRF activation, including interferon-stimulated genes. Moreover, IRF7 ranked first in the list of activated upstream regulators in the FSH-priming group. IRF7 is a master regulator of the IFNα/β immune response against viruses^43^, whose expression is crucial for myeloid cell conversion from a pro- to an anti-inflammatory state. IRF7 expression is regulated by TGFB1, and negative regulation of IRF7 is stimulated by prolonged exposure to TGFB1^44^. In granulosa cells, increased *TGFB1* is a hallmark of the activated inflammatory process, which may stimulate follicular atresia^29^. In the results obtained by IPA, TGFB1 has been predicted as an inhibited upstream regulator, which was dependent on a suppressed TGFB pathway, and the expression of *TGFB1* and the receptor *TGFR2* were lower in the FSH-priming group. Furthermore, we found that follicular atresia-like transcriptomic profile in cumulus cells, which was characterized as activation of TGFB and TP53 signalling and inhibition of angiogenesis by TSP1^45^, is suppressed in the FSH-priming group. The high expression level of *CYP19A1* observed in the FSH-priming group corroborates a healthy large follicle^30, 31^. In the natural oestrus cycle in mono-ovulatory species, only a large dominant follicle is ultimately selected for ovulation and other follicles with inhibited angiogenesis are induced into atresia by apoptosis signalling in granulosa cells; this has been attributed to the depletion of survival factors, in particular FSH^46^. Therefore, most COCs from a natural oestrus cycle ovary are derived from follicles in varying degrees of atresia. Artificial manipulation of follicular development by oestrus synchronization and FSH administration rescues small antral follicles from atresia via suppression of STAT3 signalling^47^. STAT3 was predicted as an inhibited upstream regulator in the FSH-priming group. Moreover, it has been shown that STAT3 knockout or knockdown cells display enhanced expression of IFNα/β response genes, including *OAS* and *IRF7* ^48^. Hence, the cumulus cells in cows subjected to FSH-priming may sustain cell integrity because of an induced anti-inflammation system, probably via increased *IRF7* expression resulting from inhibited TGFB1 and/or STAT3 signalling (Fig. 6a).

Apart from survival factors such as FSH, paracrine signalling from oocytes participate in regulating follicular atresia. This signalling could be triggered by OSFs such as BMP15 or cumulin via the activation of AKL6 (BMPR1B)-SMAD1/5/8, but not OSFs such as GDF9^12, 49^. In fact, BMPR1B transcripts are up-regulated in atretic follicles in cattle^50^. In sheep as a mono-ovulatory animal model, loss-of-function mutation of *BMPR1B* stimulates multi-ovulation by suppressing atresia of small antral follicles^51^. On the other hand during late folliculogenesis, BMPR1B signalling works as a key player for cumulus expansion and ovulation following the surge in gonadotrophin levels. The present study showed that although the expression levels of *BMP15* and *GDF9* in oocytes did not differ between the control and FSH-priming (Supplementary Fig. S1), *BMPR1B* expression was down-regulated in cumulus cells in the FSH-priming group. A diminished role for BMP and SMAD1/5/8 signalling in the FSH-priming group may be consistent with natural mammalian polyovulation and high fecundity being caused, at least to a certain extent, by a diminished role for BMP15 relative to GDF9^52, 53^.

Another interesting finding is that dendritic cell maturation was predicted as an inhibited pathway in cumulus cells in the FSH-priming group. Dendritic cell maturation is triggered by pathogens, tissue damage, and local inflammation^54, 55^ and the cells present in the ovarian environment^56^. The small ovarian dendritic cell population is essential for LH-stimulated up-regulation of specific ovulatory genes that are crucial for cumulus mucification and expansion and subsequent ovulation^57^. Ovulation has long been likened to an inflammation-like proses^58^. Hence, inhibition of dendritic cell maturation by FSH-priming, which may be associated with the suppression of initiation of spontaneous ovulation-like cascades before the gonadotrophin surge, probably regulates the responsiveness to intrinsic cumulus mucification and expansion needed for ovulation and acquisition of developmental competence.

In conclusion, the present study showed that cumulus cells undergoing advanced follicular growth prepare the oocyte for final maturation; in part by promotion of cell–to-cell communication and anti-inflammation as well as EGF-like peptide responsiveness, probably allowing efficient transfer of nutritional molecules from the somatic component of the follicle to the oocyte. These processes are likely to constitute important components of oocyte developmental competence (Fig. 6b). This concept and further IVM improvement may help to obtain high competence oocytes for animal production and human ART.

## Materials and Methods

### Chemical

Unless specified, all chemicals were purchased from Sigma-Aldrich (St Louis, MO, USA).

### Animal care and use

This study was approved by the Ethics Committee for the Care and Use of Experimental Animal at the National Livestock Breeding Center located in Nishigo, Japan. All animals received human care according to guideline numbers 6, 22 and 105 of the Japanese Guidelines for Animal Care and Use.

### Collection of cumulus-oocyte complexes (COCs) via ovum pick-up (OPU)

We performed 4 series of OPU throughout the present study. Four cows were used for each OPU series. As described previously^27^, COCs were collected from total of 16 pubertal Japanese Black cows of 45 to 74 months old by OPU using an ultrasound scanner (SSD-900; ALOKA, Tokyo, Japan) and 7.5-MHz convex array transducer (UST-9109P-7.5; ALOKA) with a 17-gauge stainless steel needle guide. Follicles were categorized as small (2–4 mm in diameter), medium (5–8 mm), and large (>8 mm). Follicles >2 mm in diameter were vacuum-aspirated (120 mmHg and 22 mL/min aspiration rate) through a disposable aspiration needle (COVA Needle; Misawa Medical, Tokyo, Japan).

### Experimental design and follicular growth treatment (FSH-priming)

**Figure 7:**
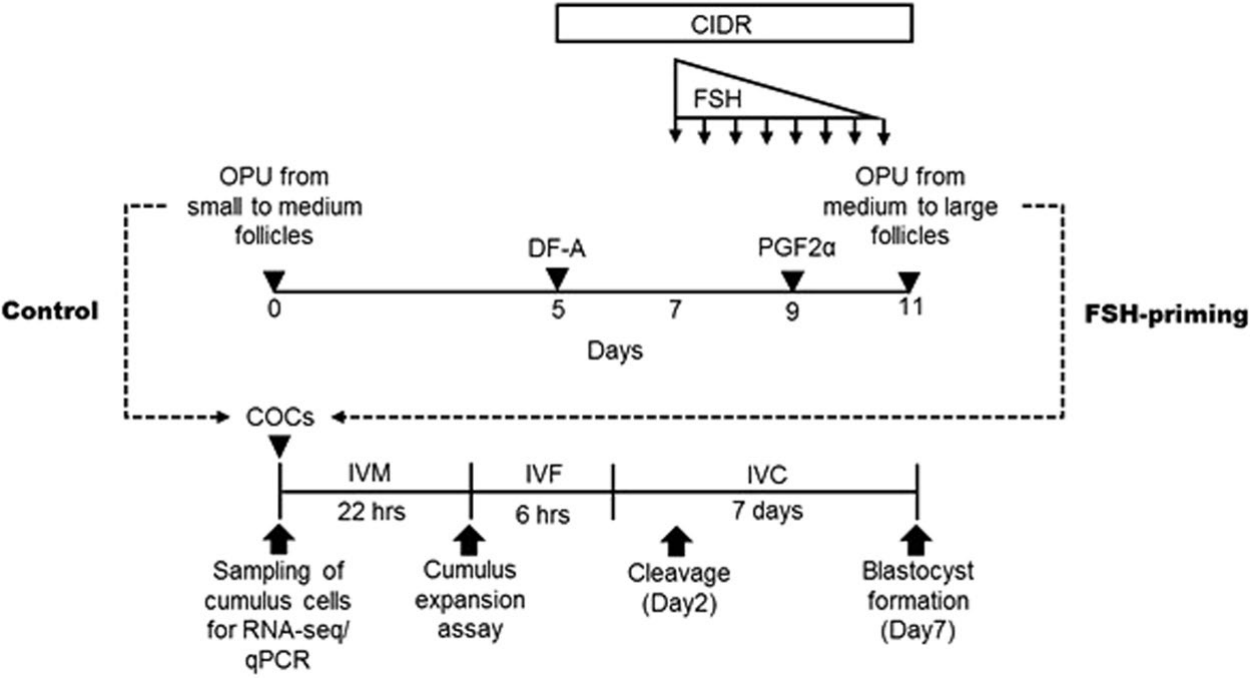
Schematic representation of experimental design. The first OPU from small to medium follicles was performed on unstimulated animals on arbitrary days of the oestrus cycle (control). Then, follicles larger than 8 mm in diameter were ablated (DF-A), and CIDRs were inserted on day 5 (the day of first OPU = day 0). The cows then received 30 mg FSH twice a day from days 7 to 10, in decreasing doses (6, 6, 4, 4, 3, 3, 3, and 2 mg) by intramuscular injection. Cloprostenol (PGF2α; 0.75 mg) was administered in the morning of day 9. The second OPU was performed 48 h after PGF2α administration (day 11), and only follicles larger than 5 mm in diameter (medium and large) were aspirated. CIDRs were removed from cows just before OPU (FSH-priming). COCs of only morphological category A and B were used for investigating cumulus expansion and developmental competence of oocytes and analysing the RNA-seq and real-time qPCR of cumulus cells.

The first OPU session (OPU without stimulation) was performed at arbitrary days of the estrous cycle (Day 0), yielding the control COCs from small and medium follicles (Fig. 7). Then on day 5, dominant follicles were ablated (DFA) by aspirating follicles larger than 8 mm in diameter (these COCs were discarded) and a progesterone-release controlled internal drug releasing (CIDR) device (CIDR 1900; Pfizer Animal Health, Hamilton, New Zealand) was inserted intravaginally. Cows then received a total of 30 mg FSH (ANTORIN-®∙10; Kyoritsu Seiyaku, Tokyo, Japan) from days 7 to 10, administered in twice daily intramuscular injections in decreasing doses (6, 6, 4, 4, 3, 3, 2 and 2 mg). PGF2α (0.75 mg; Cloprostenol; Clopromate C; Sumitomo Pharmaceuticals Co., Tokyo, Japan) was administration in the morning of day 9. The second OPU section (FSH-priming treatment) was performed 48 h after PGF2α administration (day 11), and only follicles larger than 5 mm in diameter (i.e., medium and large follicles) were aspirated. CIDR was removed from cows just before OPU^27^ (Fig. 7).

COCs morphological assessment was slightly modified from previous report^59^. COCs collected by OPU were categorized into 4 groups according to morphology of oocyte cytoplasm and cumulus cells: category A; COCs with homogenous oocyte cytoplasm and a complete, compacted, and more than 6 layers of cumulus; category B, the cumulus was smaller than in category A COCs but had 3 to 5 layers of compact cumulus cells; category C, the COCs had 1 to 2 layers of cumulus cells; category D, the cumulus was completely part absent or expanded, or oocyte cytoplasm was degenerated. In both treatment groups (control, FSH-priming), only COCs of category A and B were used in all experiments. Category A and B COCs were then subjected to either (Figure 1), (1) *in vitro* culture (end-points: cumulus expansion and blastocyst production), or (2) immediate (0 hours) cumulus cell stripping for RNA analysis (see below).

### *In vitro* maturation (IVM)

The IVM medium was HEPES-buffered TCM 199 supplemented with 4 mg/ml of fatty-acid free bovine serum albumin (BSA), 0.5 mM pyruvate and 0.1 IU/ml FSH (Follistim; MSD, Tokyo, Japan). Compacted COCs with >3 layers of cumulus cells and a homogeneous ooplasm were washed twice with the IVM medium and cultured in 100 μl IVM medium covered with paraffin oil (Paraffin Liquid; Nakalai Tesuque, Inc., Kyoto, Japan) in 35-mm Petri dishes at 38.5 °C in humidified atmosphere of 5% CO2 in air for 20 h.

### COCs expansion assay

Cumulus expansion assay that is widely used was performed as described^24^. Cumulus expansion was scored at 22 h of IVM with 100 ng/ml recombinant human amphiregulin (AREG; R&D systems, Mineapolis, MN, USA). Degree of cumulus expansion was scored into 0 to +4. A score of 0 indicate no detectable response, +1 indicating the minimum response with cells in the peripheral two layers beginning to expand, +2 indicating expansion extending inwards to several layers, +3 indicating expansion of all cumulus layers except the corona radiata cells, and +4 indicating expansion of the entire cumulus including the corona radiate cells.

### *In vitro* fertilization (IVF)

IVF was performed as reported previously^60^. At the end of IVM, ejaculated sperm samples from Japanese black bulls were thawed and then centrifuged in 3 ml of 90% Precoll solution (GE Heathcare, Uppsala, Sweden) at 750 × g for 10 min. After centrifugation, the pellet was re-suspended and centrifuged in 6 ml of sperm washing solution (Brackett and Oliphant solution, BO)^61^, supplemented with 10 mM hypotaurine (Sigma) and 4 U/mL heparin (Novo-Heparin Injection 1000; Aventis Pharma Ltd., Tokyo, Japan) at 550 × g. Then the pellet was re-suspended in sperm-washing solution and BO solution supplemented with 20 mg/ml BSA (Sigma) to achieve a final concentration of 3 × 10^6^ sperm/ml. 100 µl droplets of this suspension were aliquoted in 35-mm dishes under paraffin oil as fertilization droplets. COCs were washed twice in BO supplemented with 10 mg/ml BSA and cultured in the fertilization droplets for 6 h at 38.5 °C in 5% CO_2_ in air with saturated humidity.

### *In vitro* culture (IVC)

Charles Rosenkrans 1 medium with amino acids (CR1aa) was used for IVC medium^62^. IVC of embryos was performed at 38.5 °C in 5% O_2_, 5% CO_2_, and 90% N_2_ with saturated humidity for 7 days in 125 μl CR1aa placed in a microwell culture dish^63^. The microwell culture dish allows embryos to be cultured in an individually identifiable manner without affecting embryo density^64^. After 6 h of insemination, cumulus cells and sperm were completely removed from zygotes by pipetting with a glass pipette in IVC medium. Zygotes were placed in microwells of the culture dish.

### RNA extraction

Cumulus cells were removed from immature COCs (0 hours) by pipetting with a grass pipette in PBS. Cumulus cells were lysed in 300 µl of RTL buffer containing 10 µl/ml of 2-mercaptoethanol and stored at −80 °C. Total RNA was extracted from each sample using the RNeasy Micro Kit (Qiagen, CA, USA). Genomic DNA was removed by digestion with recombinant RNase-free DNase I (Qiagen). RNA was quantified using a NanoDrop spectrophotometer (Thermo Fisher Scientific, MA, USA). To identify oocyte specific genes with RNA sequence, RNA from the oocytes of four cows was extracted in the same manner as for cumulus cells.

### RNA sequence

Total RNA derived from four cows per treatment (one biological replicate/treatment) was used for library construction using the TruSeq Stranded mRNA LT Sample Prep Kit (Illumina, CA, USA) according to the manufacturer’s protocol. RNA purity and integrity were assessed using TapeStation 2200 (Agilent Technologies, CA, USA), and all samples had a RNA Integrity Number Equivalent (RINe) value of >8.0. The libraries were sequenced on the Illumina HiSeq. 2500 platform (Illumina) with 101-bp paired-end reads. The reads were aligned to the reference genome (NCBI UMD_3.1) using TopHat (v.2.0.13) (Trapnell *et al*. 2012) with the Refseq gene annotation. Expression levels (FPKM) of Refseq genes were calculated using Cufflinks (v. 2.2.1)^65^.

### Analysis of biofunction, upstream regulators and canonical pathways

Ingenuity pathway analysis (IPA) was used in differentially expressed genes. Lists of mRNA differentially expressed between control and FSH-priming grouped, which was defined as (FSH-priming_FPKM + 0.1)/(Control_FPKM + 0.1) ≥ 2 and FSH-priming_FRKM ≥ 1 and (FSH-priming_FPKM + 0.1)/(Control_FPKM + 0.1) ≤ 0.5 and Control_FPKM ≥ 1, were uploaded in the ingenuity package. In order to eliminate the possibility of oocyte contamination, oocyte-specific genes were deleted from the list of mRNA. Oocyte-specific genes was defined as Control_Cumulus cell_FPKM < 1 and Control_Oocyte_FPKM ≧ 10 (Supplementary Table S2). Significantly affected diseases, biofunctions, upstream regulators and canonical pathways were defined based on the *P*-value of Fischer’s exact test and the activation Z-score. The predicted upstream regulators and their downstream targets were visualized using Circos^66^.

### Comparison between transcriptomic profile of the present RNA-seq data and atretic follicular somatic cells

Present RNA-seq data were compared with microarray probe data of granulosa cells derived from well characterised bovine ovarian atretic follicles, which was reported by Hatzirodos *et al*.^29^. Statistical significance of number of overlapped genes between data sets of present RNA-seq and Hatziodos *et al*. were analyzed by Chi-squared test using R statistical software version 2.15.0 (R Foundation for Statistical Computing).

### mRNA expression by real-time RT-PCR analysis

For validation of RNA-seq data, cumulus cell mRNA expression from eight cows was analysed individually with quantitative real-time PCR as described^8^. The cumulus cell samples were from different animals that were used to generate the RNA-seq data. Beta-actin (*ACTB*) was used as the endogenous control. Total RNA (200 ng) extracted from cumulus cells from individual cows was reverse transcribed with random primers (Invitrogen, CA, USA) using Super-Script III (Invitrogen). The real-time PCR analysis was performed on a StepOne^TM^ instrument (Applied Biosystems, Foster City, CA, USA) in a 20-µl reaction volume containing 3 µl cDNA, 2.5 µl each of forward and reverse primers (Supplementary Table S1), 2 µl nuclease-free water and 10 µl of SYBR Green PCR Master Mix (Applied Biosystems). PCR reactions were performed in duplicate. Universal thermal cycling parameters (initial step of 2 min at 50 °C and 10 min at 95 °C, followed by 40 cycles of 15 s at 95 °C and 60 s at 60 °C) were used. Melting curve analysis was carried out on the real-time cycler to check the specificity of the reaction. A standard curve was generated for the genes in every PCR run by using a serial 5-fold dilution of amplified cDNA derived from cumulus cells. Results were normalized to *ACTB* and expressed relative to a mean value of control which was set at 1.

### Statistical Analysis

All data, with the exception of RNA-seq data, were analyzed using analysis of variance (ANOVA) followed by Tukey-Kramer test or Student t-test JMP (SASS). Prior to ANOVA and Student t-test, Kolmogorov-Smirnov test and Bartlett test were used for normality and homogeneity of variance, respectively. RNA-seq data was analysed using R statistical software version 2.15.0. All percentage data were arcsine transformed prior to analysis.

## Electronic supplementary material

Table S1

Table S2

Table S3

Table S4

Table S5

Table S6

Supplementary Information

## References

[1] Gilchrist RB, Thompson JG (2007). Oocyte maturation: emerging concepts and technologies to improve developmental potential *in vitro*. Theriogenology.

[2] De Vos M, Smitz J, Thompson JG, Gilchrist RB (2016). The definition of IVM is clear-variations need defining. Human reproduction (Oxford, England).

[3] Erickson GF, Wang C, Hsueh AJ (1979). FSH induction of functional LH receptors in granulosa cells cultured in a chemically defined medium. Nature.

[4] El-Hayek S, Demeestere I, Clarke HJ (2014). Follicle-stimulating hormone regulates expression and activity of epidermal growth factor receptor in the murine ovarian follicle. Proceedings of the National Academy of Sciences of the United States of America.

[5] El-Hayek S, Clarke HJ (2015). Follicle-Stimulating Hormone Increases Gap Junctional Communication Between Somatic and Germ-Line Follicular Compartments During Murine Oogenesis. Biology of reproduction.

[6] Yun SP (2012). Mechanism of PKA-dependent and lipid-raft independent stimulation of Connexin43 expression by oxytoxin in mouse embryonic stem cells. Molecular endocrinology (Baltimore, Md..

[7] Russell DL, Gilchrist RB, Brown HM, Thompson JG (2016). Bidirectional communication between cumulus cells and the oocyte: Old hands and new players?. Theriogenology.

[8] Sugimura S (2014). Amphiregulin co-operates with bone morphogenetic protein 15 to increase bovine oocyte developmental competence: effects on gap junction-mediated metabolite supply. Molecular human reproduction.

[9] Hsieh M (2007). Luteinizing hormone-dependent activation of the epidermal growth factor network is essential for ovulation. Molecular and cellular biology.

[10] Park JY (2004). EGF-like growth factors as mediators of LH action in the ovulatory follicle. Science (New York, N.Y..

[11] Shimada M, Hernandez-Gonzalez I, Gonzalez-Robayna I, Richards JS (2006). Paracrine and autocrine regulation of epidermal growth factor-like factors in cumulus oocyte complexes and granulosa cells: key roles for prostaglandin synthase 2 and progesterone receptor. Molecular endocrinology (Baltimore, Md..

[12] Mottershead DG (2015). Cumulin, an Oocyte-secreted Heterodimer of the Transforming Growth Factor-beta Family, Is a Potent Activator of Granulosa Cells and Improves Oocyte Quality. The Journal of biological chemistry.

[13] Yoshino O, McMahon HE, Sharma S, Shimasaki S (2006). A unique preovulatory expression pattern plays a key role in the physiological functions of BMP-15 in the mouse. Proceedings of the National Academy of Sciences of the United States of America.

[14] Peng J (2013). Growth differentiation factor 9:bone morphogenetic protein 15 heterodimers are potent regulators of ovarian functions. Proceedings of the National Academy of Sciences of the United States of America.

[15] Eppig JJ, Schroeder AC, O’Brien MJ (1992). Developmental capacity of mouse oocytes matured *in vitro*: effects of gonadotrophic stimulation, follicular origin and oocyte size. J Reprod Fertil.

[16] Bagg MA, Nottle MB, Armstrong DT, Grupen CG (2007). Relationship between follicle size and oocyte developmental competence in prepubertal and adult pigs. Reproduction, fertility, and development.

[17] Eppig JJ, Schultz RM, O’Brien M, Chesnel F (1994). Relationship between the developmental programs controlling nuclear and cytoplasmic maturation of mouse oocytes. Developmental biology.

[18] Lonergan P, Monaghan P, Rizos D, Boland MP, Gordon I (1994). Effect of follicle size on bovine oocyte quality and developmental competence following maturation, fertilization, and culture *in vitro*. Mol Reprod Dev.

[19] Marchal R, Vigneron C, Perreau C, Bali-Papp A, Mermillod P (2002). Effect of follicular size on meiotic and developmental competence of porcine oocytes. Theriogenology.

[20] Ritter LJ, Sugimura S, Gilchrist RB (2015). Oocyte induction of EGF responsiveness in somatic cells is associated with the acquisition of porcine oocyte developmental competence. Endocrinology.

[21] Prochazka R, Kalab P, Nagyova E (2003). Epidermal growth factor-receptor tyrosine kinase activity regulates expansion of porcine oocyte-cumulus cell complexes *in vitro*. Biology of reproduction.

[22] Sugimura S (2015). Promotion of EGF receptor signaling improves the quality of low developmental competence oocytes. Developmental biology.

[23] Wigglesworth K, Lee KB, Emori C, Sugiura K, Eppig JJ (2015). Transcriptomic diversification of developing cumulus and mural granulosa cells in mouse ovarian follicles. Biology of reproduction.

[24] Vanderhyden BC, Caron PJ, Buccione R, Eppig JJ (1990). Developmental pattern of the secretion of cumulus expansion-enabling factor by mouse oocytes and the role of oocytes in promoting granulosa cell differentiation. Developmental biology.

[25] Hendriksen PJ, Vos PL, Steenweg WN, Bevers MM, Dieleman SJ (2000). Bovine follicular development and its effect on the *in vitro* competence of oocytes. Theriogenology.

[26] Sirard MA, Picard L, Dery M, Coenen K, Blondin P (1999). The time interval between FSH administration and ovarian aspiration influences the development of cattle oocytes. Theriogenology.

[27] Sugimura S (2012). Follicular growth-stimulated cows provide favorable oocytes for producing cloned embryos. Cellular reprogramming.

[28] Hendriksen PJ (2003). Follicular dynamics around the recruitment of the first follicular wave in the cow. Biology of reproduction.

[29] Hatzirodos N (2014). Transcriptome profiling of granulosa cells from bovine ovarian follicles during atresia. BMC genomics.

[30] Hatzirodos N (2014). Transcriptome profiling of granulosa cells of bovine ovarian follicles during growth from small to large antral sizes. BMC genomics.

[31] Irving-Rodgers HF, Harland ML, Sullivan TR, Rodgers RJ (2009). Studies of granulosa cell maturation in dominant and subordinate bovine follicles: novel extracellular matrix focimatrix is co-ordinately regulated with cholesterol side-chain cleavage CYP11A1. Reproduction (Cambridge, England.

[32] Merton JS (2003). Factors affecting oocyte quality and quantity in commercial application of embryo technologies in the cattle breeding industry. Theriogenology.

[33] Mikkelsen AL, Lindenberg S (2001). Benefit of FSH priming of women with PCOS to the *in vitro* maturation procedure and the outcome: a randomized prospective study. Reproduction (Cambridge, England.

[34] Pan H, O’Brien MJ, Wigglesworth K, Eppig JJ, Schultz RM (2005). Transcript profiling during mouse oocyte development and the effect of gonadotropin priming and development *in vitro*. Developmental biology.

[35] Richani D, Sutton-McDowall ML, Frank LA, Gilchrist RB, Thompson JG (2014). Effect of epidermal growth factor-like peptides on the metabolism of *in vitro*- matured mouse oocytes and cumulus cells. Biology of reproduction.

[36] Chen J (2013). Somatic cells regulate maternal mRNA translation and developmental competence of mouse oocytes. Nature cell biology.

[37] Gebhardt KM, Feil DK, Dunning KR, Lane M, Russell DL (2011). Human cumulus cell gene expression as a biomarker of pregnancy outcome after single embryo transfer. Fertility and sterility.

[38] Wathlet S (2011). Cumulus cell gene expression predicts better cleavage-stage embryo or blastocyst development and pregnancy for ICSI patients. Human reproduction (Oxford, England.

[39] Khan DR, Guillemette C, Sirard MA, Richard FJ (2015). Transcriptomic analysis of cyclic AMP response in bovine cumulus cells. Physiological genomics.

[40] Gilchrist RB (2016). Oocyte maturation and quality: role of cyclic nucleotides. Reproduction (Cambridge, England.

[41] Dieci C (2013). The effect of cilostamide on gap junction communication dynamics, chromatin remodeling, and competence acquisition in pig oocytes following parthenogenetic activation and nuclear transfer. Biology of reproduction.

[42] Li HJ (2016). Extending prematuration with cAMP modulators enhances the cumulus contribution to oocyte antioxidant defence and oocyte quality via gap junctions. Human reproduction (Oxford, England.

[43] Honda K (2005). IRF-7 is the master regulator of type-I interferon-dependent immune responses. Nature.

[44] Cohen M (2014). Chronic exposure to TGFbeta1 regulates myeloid cell inflammatory response in an IRF7-dependent manner. The EMBO journal.

[45] Thomas FH, Wilson H, Silvestri A, Fraser HM (2008). Thrombospondin-1 expression is increased during follicular atresia in the primate ovary. Endocrinology.

[46] Webb R (2003). Mechanisms regulating follicular development and selection of the dominant follicle. Reproduction (Cambridge, England) Supplement.

[47] Ilha GF (2015). Lack of FSH support enhances LIF-STAT3 signaling in granulosa cells of atretic follicles in cattle. Reproduction (Cambridge, England.

[48] Wang WB, Levy DE, Lee CK (2011). STAT3 negatively regulates type I IFN-mediated antiviral response. Journal of immunology (Baltimore, Md.: 1950.

[49] Fenwick MA (2013). Investigations of TGF-beta signaling in preantral follicles of female mice reveal differential roles for bone morphogenetic protein 15. Endocrinology.

[50] Gasperin BG (2014). Expression of receptors for BMP15 is differentially regulated in dominant and subordinate follicles during follicle deviation in cattle. Anim Reprod Sci.

[51] Feary ES (2007). Patterns of expression of messenger RNAs encoding GDF9, BMP15, TGFBR1, BMPR1B, and BMPR2 during follicular development and characterization of ovarian follicular populations in ewes carrying the Woodlands FecX2W mutation. Biology of reproduction.

[52] Crawford JL, McNatty KP (2012). The ratio of growth differentiation factor 9: bone morphogenetic protein 15 mRNA expression is tightly co-regulated and differs between species over a wide range of ovulation rates. Molecular and cellular endocrinology.

[53] Mcnatty KP, Juengel JL, Pitman JL (2014). Oocyte-somatic cell interactions and ovulation rate: effects of oocyte quality and embryo yield. Reprod Biol Insights.

[54] Palucka AK, Banchereau J, Blanco P, Pascual V (2002). The interplay of dendritic cell subsets in systemic lupus erythematosus. Immunology and cell biology.

[55] Banchereau J, Steinman RM (1998). Dendritic cells and the control of immunity. Nature.

[56] Heesters BA, Myers RC, Carroll MC (2014). Follicular dendritic cells: dynamic antigen libraries. Nature reviews. Immunology.

[57] Cohen-Fredarow A (2014). Ovarian dendritic cells act as a double-edged pro-ovulatory and anti-inflammatory sword. Molecular endocrinology (Baltimore, Md..

[58] Espey LL (1980). Ovulation as an inflammatory reaction–a hypothesis. Biology of reproduction.

[59] Stojkovic M (2001). Mitochondrial distribution and adenosine triphosphate content of bovine oocytes before and after *in vitro* maturation: correlation with morphological criteria and developmental capacity after *in vitro* fertilization and culture. Biology of reproduction.

[60] Sugimura S (2012). Promising system for selecting healthy *in vitro*-fertilized embryos in cattle. PloS one.

[61] Brackett BG, Oliphant G (1975). Capacitation of rabbit spermatozoa *in vitro*. Biology of reproduction.

[62] Rosenkrans CF, Zeng GQ, G. T. MC, Schoff PK, First NL (1993). Development of bovine embryos *in vitro* as affected by energy substrates. Biology of reproduction.

[63] Sugimura S (2010). Time-lapse cinematography-compatible polystyrene-based microwell culture system: a novel tool for tracking the development of individual bovine embryos. Biology of reproduction.

[64] Sugimura S (2013). Effect of embryo density on *in vitro* development and gene expression in bovine *in vitro*-fertilized embryos cultured in a microwell system. The Journal of reproduction and development.

[65] Trapnell C (2012). Differential gene and transcript expression analysis of RNA-seq experiments with TopHat and Cufflinks. Nature protocols.

[66] Krzywinski M (2009). Circos: an information aesthetic for comparative genomics. Genome research.

