# Supplementary material for: Transcriptomic signature of the follicular somatic compartment surrounding an oocyte with high developmental competence
Source: Sci Rep. 2017 Jul 28;7:6815. doi: 10.1038/s41598-017-07039-5 (PMC5533789; doi:10.1038/s41598-017-07039-5)
Supplement: Supplementary file 7 — Supplementary Information [file 41598_2017_7039_MOESM7_ESM.doc]

**Transcriptomic signature of follicular somatic component surrounding oocyte with high developmental competence**

Satoshi Sugimura^1,*+^, Norio Kobayashi^2,+^, Hiroaki Okae^3^, Tadayuki Yamanouchi^4^, Hideo Matsuda^4^, Takumi Kojima^1^, Akira Yajima^1^, Yutaka Hashiyada^4^, Masahiro Kaneda^5^, Kan Sato^1^, Kei Imai^6^, Kentaro Tanemura^2^, Takahiro Arima^3^, Robert B Gilchrist^7^

^1^Department of Biological Production, Tokyo University of Agriculture and Technology, Tokyo 183-8509, Japan

^2^Laboratory of Animal Reproduction and Development, Graduate School of Agricultural Science, Tohoku University, Miyagi 981-8555, Japan

^3^Department of Informative Genetics, Environment and Genome Research Center, Graduate School of Medicine, Tohoku University, Miyagi 980-8575, Japan

^4^National Livestock Breeding Center, Fukushima 961-8511, Japan

^5^Divition of Animal Life Science, Tokyo University of Agriculture and Technology, Tokyo 183-8509, Japan

^6^Department of Sustainable Agriculture, Rakuno Gakuen University, Hokkaido 069-8501, Japan

^7^Discipline of Obstetrics & Gynaecology, School of Women's & Children's Health, University of New South Wales, Sydney 2052, Australia

^*^Corresponding author: Satoshi Sugimura

Department of Biological Production, Tokyo University of Agriculture and Technology, 3-5-8 Saiwai-cho Fuchu-shi, Tokyo 183-8509, Japan

Tel: +81 (42) 367 5819; E-mail: satoshis@cc.tuat.ac.jp

^+^These authors contributed equally to this work


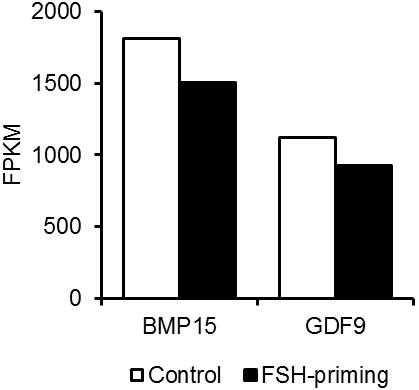


**Supplementary Figure S1.** RNA-seq analysis of oocytes from both unpriming and FSH priming cows for oocyte-secreted factors.

mRNA expression level of the oocytes derived from four donor cows without (control) or with FSH-priming were examined by RNA-seq. Expression level shown by FPKM.

**Supplementary Table S1**. Sequences of primers used for real-time RT-PCR.

**Supplementary Table S2.** List of oocyte specific genes.

**Supplementary Table S3**. List of gene expression in cumulus cells derived from donor cows without (control) or with FSH-priming.

**Supplementary Table S4**. List of diseases and biofunctions in cumulus cells derived from donor cows with FSH-priming.

**Supplementary Table S5**. List of upstream regulators in cumulus cells derived from donor cows with FSH-priming.

**Supplementary Table S6**. Llist of canonical pathways in cumulus cells derived from donor cows with FSH-priming.
